# Peripartum cardiomyopathy: a comprehensive and contemporary review

**DOI:** 10.1007/s10741-024-10435-5

**Published:** 2024-09-30

**Authors:** Farai Russell Sigauke, Hopewell Ntsinjana, Nqoba Tsabedze

**Affiliations:** 1 Division of Cardiology, Department of Internal Medicine, School of Clinical Medicine, Faculty of Health Sciences, University of the Witwatersrand, Johannesburg, 2193 South Africa; 2 Cardiology Unit, Nelson Mandela Children’s Hospital, 6 Jubilee Road, Johannesburg, 2193 South Africa

**Keywords:** Peripartum cardiomyopathy, Pathophysiology, Biomarkers, Management, Novel approaches

## Abstract

Cardiovascular disease is a major non-communicable disease globally, with increasing prevalence, posing a significant public health challenge. It is the leading non-obstetric cause of perinatal morbidity and mortality, with a substantial number of cardiac fatalities occurring in individuals without any known pre-existing cardiovascular disease. Peripartum cardiomyopathy is a type of de novo heart failure that occurs in pregnant women in the late stages of pregnancy or following delivery. Despite extensive research, diagnosing and managing peripartum cardiomyopathy remains challenging, resulting in significant morbidity and mortality. Recent advancements and novel approaches have been made to better understand and manage peripartum cardiomyopathy, including molecular and non-molecular biomarkers, genetic predisposition and risk prediction, targeted therapies, multidisciplinary care, and improved patient education. This narrative review provides a comprehensive overview and new perspectives on peripartum cardiomyopathy, covering its epidemiology, updated pathophysiological mechanisms, diagnosis, management, and future research directions for healthcare professionals, researchers, and clinicians.

## Background

Cardiovascular disease (CVD) is the leading non-communicable disease worldwide, and its burden is rising, becoming a significant global public health issue [1–3]. It is the primary non-obstetric cause of perinatal morbidity and mortality, with up to 78% of cardiac fatalities having no known pre-existing CVD [2–4]. Peripartum cardiomyopathy (PPCM) is a pregnancy-associated heart failure (HF) that mainly affects childbearing women, resulting in increased disability-adjusted life years and constitutes a significant financial strain to the healthcare systems, especially in low- and middle-income countries [5–13]. Globally, the mortality rates of PPCM range from 7 to 15% [6, 14–17]. PPCM diagnosis and management continue to pose significant challenges despite extensive research. Notably, there have been several recent substantial advancements and novel approaches to understanding and managing PPCM [5–7]. These include molecular and non-molecular biomarkers, genetic predisposition, risk prediction, targeted therapies, multidisciplinary care, and improved patient education. This narrative review provides a comprehensive understanding of PPCM, including its history, mechanisms, diagnosis, and management, and highlights future research directions for healthcare professionals, researchers, and clinicians.

## History of definition of peripartum cardiomyopathy

Peripartum cardiomyopathy, also known as postpartum cardiomyopathy or pregnancy-related HF, has had various clinical definitions and diagnostic criteria across regional and national health organizations [5, 12, 15–19]. In the late eighteenth century, Virchow and colleagues noted a relationship between pregnancy and HF [20–22]. Fraser (1935) demonstrated that HF was one of the leading causes of maternal mortality, not due to obstetric complications [23, 24]. Subsequently, in 1936, Hull and Hidden were the first to document pregnancy-related HF in New Orleans, labelling the condition “toxic” postpartal heart disease [25]. The term PPCM was introduced by Demakis and Rahimtoola in 1971 [26].

The National Heart Lung and Blood Institute (NHLBI) and The Office of Rare Diseases workshop coined the first universally accepted definition of PPCM in 2000. The definition comprised of four criteria: the development of cardiac failure in the last month of pregnancy or 5 months after delivery in the absence of an identifiable cause of cardiac failure, the absence of recognizable structural heart disease before the last month of pregnancy, and left ventricular dysfunction demonstrated by classic echocardiographic criteria, such as left ventricular ejection fraction < 45%, Motion-mode fractional shortening < 30%, or left ventricular end-diastolic dimension > 2.7 cm/m^2^ [26, 27].

In 2010, the Study group of Postpartum Cardiomyopathy of the European Society of Cardiology (ESC) proposed the following definition: “PPCM is an idiopathic cardiomyopathy presenting with heart failure secondary to left ventricular systolic dysfunction towards the end of pregnancy or in the months following delivery, where no other cause of heart failure is found. It is a diagnosis of exclusion. The left ventricle may not be dilated, but the ejection fraction is nearly always reduced below 45%” [28, 29]. Bauersachs and colleagues, in the ESC position paper 2019, simplify the definition to a criterion of three: HF secondary to left ventricular systolic dysfunction with LVEF < 45%, occurrence towards the end of pregnancy or in the months following delivery, and no other identifiable cause of heart failure. The left ventricle does not necessarily need to be dilated [5].

Clinicians and researchers worldwide currently use the ESC 2019 simplified case definition of PPCM [5, 30]. A broader proposed definition includes two additional groups: early PPCM, diagnosed as early as the first month of pregnancy until the ninth month, and late PPCM, diagnosed from 6 to 12 months post-delivery [31]. The current definition aims to clarify PPCM, enabling early diagnosis and management, thus reducing feto-maternal morbidity and mortality.

## Epidemiology

Peripartum cardiomyopathy is a universal condition with an unknown true incidence and prevalence with substantial variance in rates observed between and within countries. Worldwide, the estimated incidence rate is 1:2000, ranging from 1:300 in rural Haiti to 1:20,000 in Japan [6, 12, 16–19]. The reason for the variations between geographic locations is unknown but might be associated with ethnicity and socioeconomic factors [13]. According to a study conducted by Brar and colleagues in the USA, the condition’s overall incidence rate was 1 in 4025 live births [32]. The study also revealed that African Americans had the highest incidence rate, seven times greater than Hispanics and almost three times higher than Caucasians [8, 32]. Sub-Saharan Africa has the highest disease burden globally, with Nigeria having the highest incidence rates (1:100 live births) among the Hausa-Fulani community in Kano [8, 32]. This was believed to be linked to the cultural practice of sleeping on hot mud beds and consuming high-salt pap after childbirth. However, Sanderson and associates, with the use of echocardiography, noted that a significant number of patients were misdiagnosed as PPCM but had high-output heart failure with preserved ejection fraction. Furthermore, in the PEACE registry, this practice did not reach statistical significance [13, 33, 34].

South Africa has emerged as a global lighthouse in PPCM clinical research. In 1995, in Durban, South Africa, Desai reported a PPCM prevalence rate of 1:1000 live births, while an earlier study in Johannesburg by Seftel and colleagues described a rate of 1:3000 [35, 36]. In a 4-year cohort of 38 patients followed-up at Klerksdorp/Tshepong Hospital from 2011 to 2014, Sigauke and colleagues reported an incidence rate of 1: 1000, which mirrors rates reported in the Heart of Soweto Study in Johannesburg, which included approximately 200 women with PPCM [14, 37, 38]. However, it is worth noting that previous studies have been limited by small sample sizes and conducted in single centers. The exact incidence of PPCM in other continents, namely, Asia, Australia, and Europe, remains unknown, and ongoing worldwide registries like the EURObservational Research Programme and local epidemiological studies will answer some questions [6, 7].

Peripartum cardiomyopathy is prevalent across all age groups in women; nonetheless, it demonstrates greater prevalence and is linked to more adverse outcomes among women at the lower and upper ends of the reproductive age spectrum (< 20 and > 35 years) [5, 7, 8, 13, 16, 32]. The mean age in worldwide large registries and studies ranges from 28.9 to 33 years (IPAC study, PEACE registry, Global ESC EORP and German study) (7–9,13). The global variance can be attributed to differences in ethnicity, environment, and timely access to diagnosis. There is a higher prevalence of undiagnosed diseases in older women, which may act as confounders [5, 39–41]. Contrary to previous reports, the PEACE study in Nigeria noted a high incidence of PPCM among younger women [13]. However, the study’s small sample size, lack of power, and geographic and economic variation could explain the discrepancy. Approximately 19% of cases are diagnosed during the final month of pregnancy, while 75% of PPCM patients are diagnosed within the first month after delivery. Out of this group, 45% are diagnosed during the first week following childbirth [32, 42].

The global incidence rate of peripartum cardiomyopathy (PPCM) is expected to be determined through the standardized case definition, increased disease awareness, improved accessibility to echocardiography, and various ongoing registries. Due to the acute nature of PPCM, the prevalence rate is closely associated with the incidence rate.

## Aetio-pathophysiology

The exact aetio-pathophysiology of PPCM is unknown [12, 16, 17]. Numerous clinical studies and national registries have extensively investigated various factors and mechanisms in the pathophysiology of PPCM. Among these mechanisms, the vasculo-hormonal theory is believed to be the most plausible explanation for the etiology of PPCM (Table 1) [16, 17]. The vascular model of PPCM focuses on the cardiac inability to adapt to the haemodynamic changes and stresses that occur during pregnancy and the peripartum period, resulting in cardiomyocyte release of vasoactive molecules that eventually cause angiogenic imbalance and vascular dysfunction. The “two-hit” hypothesis suggests that in genetically susceptible individuals, multiple insults to the myocardium before or during pregnancy trigger an inflammatory response during gestation and postpartum, leading to impaired myocardial function [16–19]. There is a temporal mismatch between the peak of haemodynamic stress in the second trimester and the onset of PPCM in the third or fourth trimester (12 weeks postpartum), suggesting the involvement of other probable factors. These factors include viral infection, autoimmunity, hormonal imbalance, and nutritional deficiencies like selenium [17].

**Table 1:** Several mechanisms in the vasculo-hormonal model in the pathogenesis of PPCM

Inflammatory response and autoimmune mechanisms	Inflammation: Pregnancy is associated with changes in the immune system’s ability to tolerate the fetus. PPCM may result from an exaggerated inflammatory response, leading to myocardial inflammation and damage. Inflammation can be triggered or exacerbated by viral infections
	Autoimmunity: Evidence suggests an autoimmune process may be involved, in which the body’s immune system mistakenly attacks the heart tissue
Prolactin and oxidative stress	Prolactin fragments: In late pregnancy and postpartum, high prolactin levels, a hormone produced by the pituitary gland, are normal. In postpartum cardiomyopathy (PPCM), a 16-kDa prolactin fragment created by cathepsin D cleavage is believed to cause endothelial damage, inflammation, and cardiomyocyte apoptosis
	Oxidative stress: Increased oxidative stress in the peripartum period can contribute to cardiac dysfunction. Oxidative stress can lead to the generation of reactive oxygen species (ROS), which damage cardiac cells and impair their function
Genetic predisposition	Genetic factors: Genetic predisposition plays a role in PPCM. Mutations in genes encoding sarcomeric proteins, cytoskeletal proteins, and other cardiac-related genes have been identified in some PPCM patients. These genetic mutations may make the heart more vulnerable to stress during pregnancy
Hemodynamic stress	Volume overload: Pregnancy is associated with increased plasma volume and cardiac output to meet the metabolic demands of the mother and fetus. This increased workload can unmask underlying myocardial dysfunction or exacerbate pre-existing heart conditions
	Left ventricular remodelling: Changes in the heart’s structure and function during pregnancy, such as left ventricular hypertrophy and remodelling, may predispose some women to PPCM
Angiogenic imbalance	Imbalance of angiogenic factors: Pregnancy involves significant changes in angiogenic factors, such as vascular endothelial growth factor (VEGF) and placental growth factor (PlGF). An imbalance between pro-angiogenic and anti-angiogenic factors can lead to endothelial dysfunction and cardiac dysfunction
Metabolic and hormonal changes	Metabolic stress: The metabolic changes of pregnancy, including glucose and lipid metabolism alterations, can place additional stress on the heart
	Hormonal changes: Hormones such as estrogen and progesterone, which are elevated during pregnancy, affect cardiovascular function and may contribute to PPCM in susceptible individuals

The several vasculo-hormonal theory mechanisms implicated in the pathogenesis of peripartum cardiomyopathy (PPCM) interact synergistically in genetically predisposed individuals, contributing to myocardial dysfunction

**Fig. 1:**
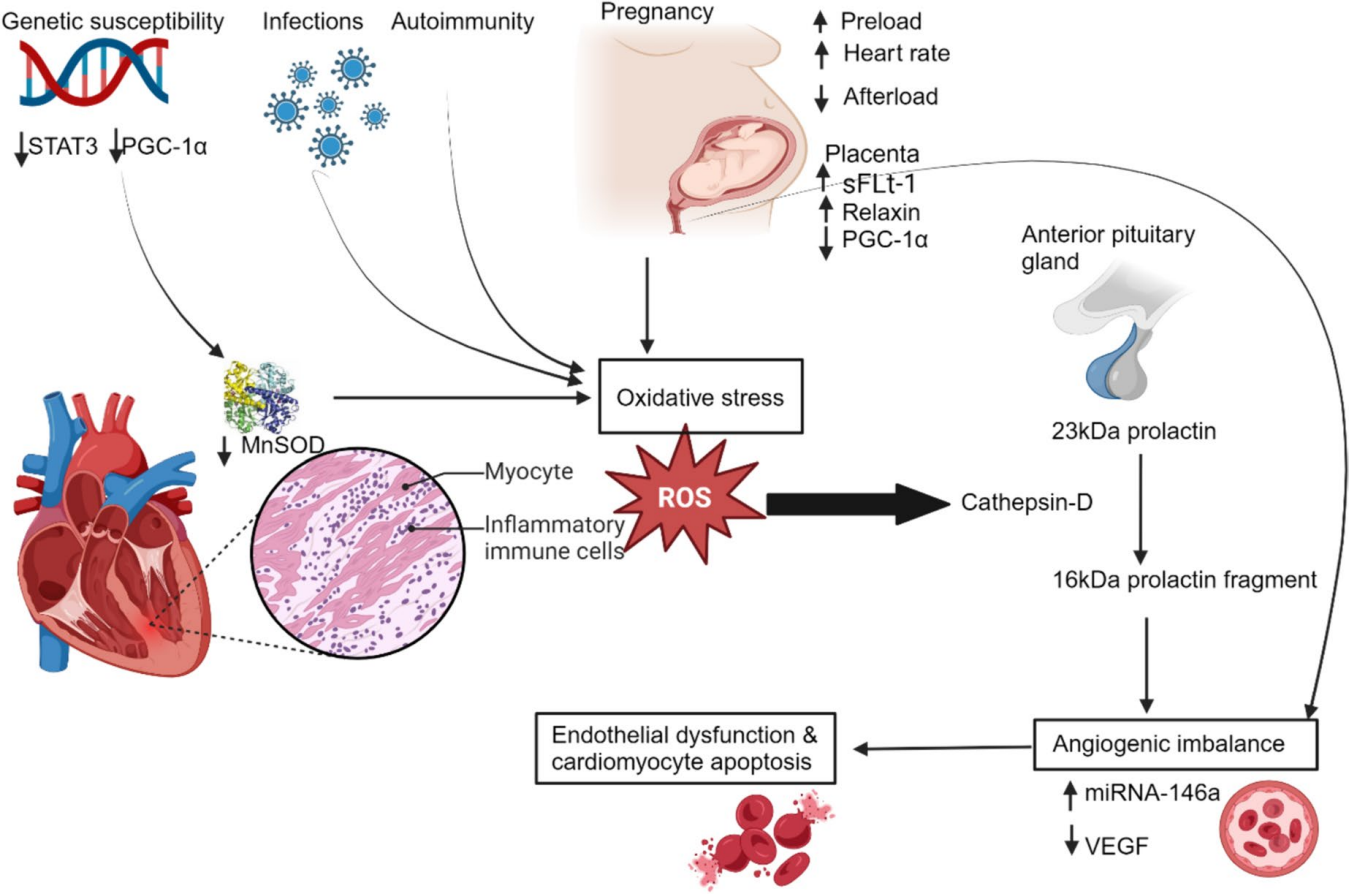
Novel pathophysiological mechanisms of peripartum cardiomyopathy. Oxidative stress triggered by pregnancy, genetic susceptibility, infections, and autoimmune factors promotes the release of cathepsin-D from the cardiomyocyte, which promotes the proteolytic cleavage of 23 kDa prolactin to an anti-angiogenic form known as 16 kDa prolactin (also known as vasoinhibin) leading to angiogenic imbalance and, subsequently, endothelial dysfunction and cardiomyocyte apoptosis. kDa, kilodalton; miRNA-146a, micro-ribonucleic acid 146a; MnSOD, manganese superoxide dismutase; PGC-1α, peroxisome proliferator-activated receptor gamma coactivator 1-alpha; ROS, reactive oxygen species; sFLt-1, soluble Fms-like tyrosine kinase 1; STAT3, signal transducer and activator of transcription 3; VEGF, vascular endothelial growth factor. Created with BioRender.com

Lately, there has been a paradigm shift in the critical processes hypothesized in the pathophysiology of PPCM [5, 17, 43]. Vascular angiogenic imbalance, increased oxidative stress, and inflammation are the principal mechanisms supported by in vivo and ex vivo (knock-out STAT mice with PPCM) evidence [44–46]. The development of PPCM through vascular impairment arises from two separate pathophysiological pathways involving the pituitary and placenta, resulting in an angiogenic imbalance (Fig. 1). The first pathway involves 16 kDa prolactin (PRL), nuclear factor-kappa B(NFκB), and microRNA-146a. The second pathway is regulated by the balance between the placental-derived anti-angiogenic factor soluble fms-like tyrosine kinase-1 (sFlt1) and pro-angiogenic factor vascular endothelial growth factor (VEGF) [16, 17, 43].

In the first pathway, the increased oxidative stress state during pregnancy reduces the signal transducer and activator of the transcription 3 (STAT3) factor. This reduction curtails the expression of the reactive oxygen species (ROS) scavenger, manganese superoxide dismutase (MnSOD) [44]. The decreased level of MnSOD results in the accumulation of ROS, leading to the escalation, activation, and secretion of peptidase cathepsin D from the cardiomyocytes into the circulation. During the peripartum period, the anterior pituitary gland secures elevated levels of the full-length lactation hormone 23 kDa PRL. Cathepsin D cleaves 23 kDa PRL in the cardiac vasculature into the cardiotoxic and vasculotoxic fragment 16 kDa PRL (vasoinhibin), which acts on urokinase-type plasminogen activator receptor (uPAR) to induce endothelial damage, impairing cardiomyocyte metabolism, resulting in apoptosis. Vasoinhibin is a driver of PPCM through the transcription factor, nuclear factor kappa-B (NFκB), that upregulates endothelial expression of microRNA-146a [5, 17, 19]. MicroRNA-146a, an endothelial secretome, causes cardiomyocyte dysfunction and apoptosis by blocking multiple pathways involving Erbb4, Nras and Notch1[19]. Vasoinhibin, in concert with interferon-γ (IFN-γ) derived from inflammation, induces cardiac hypertrophy orchestrated by protein kinase B (Akt) [44].

In the second pathway, the placental expression of the pro-angiogenic factor VEGF is downregulated through two mechanisms [16]. In the peripartum period, the placenta secretes several vasoactive molecules. The secretion of peroxisome proliferator-activated receptor gamma 1-alpha coactivator (PGC1α), a potent regulator of angiogenesis, is downregulated. Additionally, there is an increase in the production of the anti-angiogenic sFlt1, which leads to inadequate upregulation of VEGF, worsening the angiogenic milieu of PPCM [12, 19]. Studies have shown that the suppression of PGC1α in murine hearts is associated with sFlt1-induced cardiomyopathy. Elevated levels of sFlt1 have also been linked to disease severity and a poor prognosis [16, 46]. During this period, hormonal levels of progesterone and activin A increase while levels of vasoprotective relaxin 2 decrease. Progesterone regulates cardiac energetics by suppressing glucose metabolism, cardiomyocyte hypertrophy, and dysfunction [17]. Activin A, a transforming growth factor-beta (TGF-β) superfamily member, promotes cardiomyocyte inflammation, fibrosis, and remodelling in PPCM, resulting in injury and dysfunction. Activin A levels may become part of the diagnostic and prognostic toolkit for managing PPCM, an emerging area of interest [47–49].

Several risk factors are associated with a preponderance of disease progression, rendering the host vulnerable to PPCM. Traditionally, predisposing risk factors have been stratified as possible, probably, and emerging [14]. These factors include multi-parity, twin gestation, extended breastfeeding, increased maternal age, selenium deficiency, smoking, alcohol, illicit drug use, low body mass index (BMI) < 18.5 kg/m^2^ (underweight), African ancestry, family history of disease, and gestational hypertensive disorders [13, 14, 18]. Individuals who have experienced PPCM in the past are likely to experience a relapse or deterioration in subsequent pregnancies [42].

Recent research has focused on emerging risk factors, especially gestational hypertensive disorders such as gestational hypertension, pre-eclampsia, and eclampsia [29]. This disease group is associated with significant cardiovascular morbidity and mortality in addition to PPCM [19, 43]. In a cohort of patients with hypertensive heart failure of pregnancy (HHFP) and idiopathic PPCM, significant differences in clinical presentation and outcomes were noted, with the possibility that HHFP may not fit the case definition of PPCM; suffice to say this has not translated to clinical research and practice [50, 51]. Many cohorts of PPCM patients have noted a high prevalence of hypertensive disorders and pre-eclampsia in pregnancy, suggesting that this group of diseases predisposes them to develop PPCM. Notably, gestational hypertensive heart disease and PPCM present a clinical conundrum as they manifest similar symptomology, yet they have divergent management, including anti-hypertensive and anti-failure therapies [52].

In 1963, Pierce and colleagues were the first to report family clustering of PPCM cases and the possibility of genetic predisposition in the pathogenesis [53]. Subsequently, multiple studies have supported the role of genetics in up to 15% of patients [54]. Fett and colleagues in Haiti reported cases of PPCM in a mother and her daughter, further supporting the role of genetics in the manifestation of the disease [55]. Additional evidence of familial occurrence includes variable prevalence in different regions and ethnic groups [56]. Dewi and Nugroho categorized genetically associated PPCM into three groups: PPCM as a subgroup of familial dilated cardiomyopathy, PPCM associated with titin (TTN), and PPCM in women with carrier X-linked cardiomyopathy. Worldwide, the giant sarcomeric protein-coding gene with titin (TTN) truncating mutation has been the predominant genetic contributor to developing PPCM, among others, beta-myosin heavy chain, myosin-binding protein C (MYBPC3), lamin A/C, or sodium voltage-gated channel alpha subunit 5 (SCN5A) [57, 58]. X-linked cardiomyopathy includes Duchenne muscular dystrophy (DMD), Becker muscular dystrophy (BMD), and lysosomal-associated membrane protein (LAMPs) gene mutations [56, 57].

The “two-hit hypothesis” theory proposes that in gene-positive, phenotype-negative women without clinical symptoms before pregnancy, the physiological stress caused by the anti-vascular and hormonal effects of pregnancy and delivery may unmask the concealed cardiomyopathy [57]. Although further research is warranted, the advent of genome-wide association studies (GWAS) and molecular techniques has provided a better understanding of how genetics, proteomes, non-coding ribonucleic acids (RNA), heat shock proteins, and chaperones may play a role in the pathogenesis of PPCM [58–60]. However, more than 90% of individuals with genetic variants do not develop PPCM, suggesting the implication of extra-genetic factors [15].

It is important to note that the exact mechanisms and triggers of PPCM are not yet fully understood despite the valuable insights provided by the theories above. Nevertheless, ongoing research is diligently working to unravel the intricate interplay of various factors contributing to PPCM. The goal is to improve diagnosis and treatment approaches confidently.

## Biomarkers of peripartum cardiomyopathy (molecular vs. non-molecular)

Patients with PPCM usually present in acute HF and are investigated similarly to other cardiomyopathies as it is a diagnosis of exclusion [5, 61–63]. This daunting task delays the diagnosis, hence the need for biomarkers to tease out PPCM from its mimics, such as HHP, myocarditis, pulmonary embolus, and pre-existing dilated cardiomyopathy. Biomarkers offer a cost-effective, non-invasive, and easily measurable method for diagnosing PPCM and establishing prognosis while also providing crucial information on complex pathophysiology [60, 64]. It is crucial to research biomarker screens during the antepartum and postpartum periods due to the distinct kinetic patterns of specific hormones, growth factors, and enzymes [5, 29, 65]. In this review, biomarkers are categorized as molecular and non-molecular.

### Molecular biomarkers

**Fig. 2:**
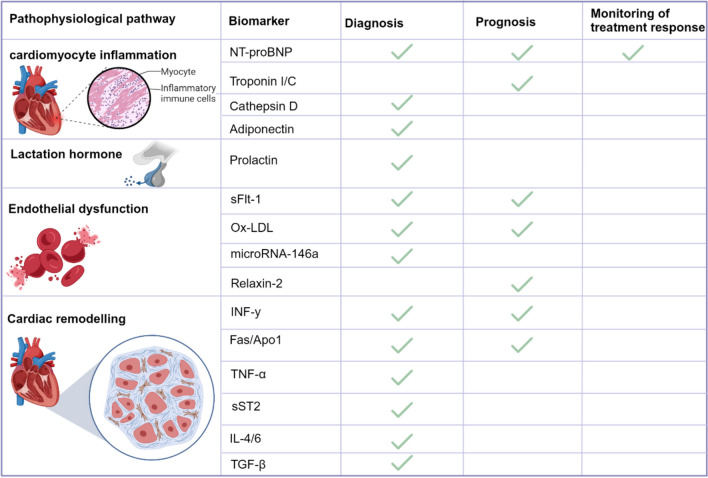
Summary of the clinical utility of biomarkers in peripartum cardiomyopathy. Several established and novel biomarkers involved in the pathophysiology of peripartum cardiomyopathy are used in diagnosing, prognosis, and monitoring treatment response. The green tick indicates the purpose for which a biomarker is used in clinical practice. Fas/Apo1, Fas cell surface death receptor/ apoptosis antigen 1; IL-4/6, interleukin 4 and 6; INF-y, interferon-gamma; microRNA-146a, microribonucleic acid-146a; NT-proBNP, N-terminal Pro B-type natriuretic peptide; Ox-LDL, oxidized low-density lipoproteins; sFlt-1, soluble Fms-like tyrosine kinase 1; sST2 soluble suppression of tumourigenicity-2; TGF- β, transforming growth factor- beta; TNF-α, tumor necrosis factor-alpha. Created with BioRender.com

Recently, numerous novel macromolecules have been evaluated for the diagnosis, prognostication, and treatment of HF in the general population, with the potential of being extrapolated to PPCM [66, 67]. These novel HF biomarkers are stratified according to their role in the pathogenesis of PPCM, namely, cardiomyocyte inflammation, fibrosis, hypertrophy, and apoptosis (Fig. 2).

Biomarkers such as troponins, brain natriuretic peptide (BNP), N-terminal pro-BNP (NT-proBNP), and mid-regional pro-atrial natriuretic peptides (MR-proANP) are routinely used in the diagnosis and monitoring of HF, including PPCM [15, 62, 63]. Natriuretic peptides (NP) are produced in response to atrial stretch and have high specificity for HF in general [15, 68]. Natriuretic peptides, especially NT-proBNP, are well-established in the diagnosis of PPCM in the acute setting as they have a very high negative predictive value when using low cut-off levels (i.e., BNP < 100 pg/ml, NT-proBNP < 300 pg/ml, and MR-proANP < 120 pmol/l) [5, 62, 63]. Hoevelmann and colleagues demonstrated that NT-proBNP > 900 pg/ml at diagnosis was associated with a poor prognosis for left ventricular recovery in a cohort of 42 women [69]. The elevation in NP and troponin levels lacks specificity for PPCM and may also be present in other acute chest syndromes, both cardiac and non-cardiac, including myocardial ischemia, tachyarrhythmia, pulmonary embolism, and preeclampsia [15].

Numerous biomarkers have been identified as potential indicators of PPCM, including microRNA-146a, cathepsin D, 16 kDa-PRL, interferon-γ, asymmetric dimethylarginine (ADMA), and sFlt1. Ongoing evaluation is being conducted to assess their accuracy in diagnosing PPCM. Additionally, other biomarkers such as soluble suppression of tumorigenicity 2 (sST2), Galectin 3 (Gal-3), relaxin, VEGF, growth-differentiation factor-15 (GDF-15), adrenomedullin (ADM), long noncoding RNA, and heat shock proteins are currently under investigation for their clinical significance in routine medical practice. These biomarkers can potentially improve diagnostic capabilities and advance medical care in PPCM management.[16–19, 43, 70–72].

### Non-molecular biomarkers

This category of biomarkers includes physical characteristics, histological and imaging (radiological) parameters measured as indicators of normal biological processes, pathological processes, or responses to therapeutic intervention [73]. The diagnosis of PPCM requires a high index of suspicion as it can present anywhere in the continuum, from insidious nonspecific symptoms of fatigue, malaise, and congestion, which can be confused as regular physiological changes of pregnancy to dramatic, acute, severe decompensated cardiac failure in extremis [5]. The clinician must obtain a detailed history and focused physical examination from the outset. Libhaher and colleagues noted that common physical characteristics like hypotension and resting tachycardia were predictors of poor outcomes [74]. Subjective but useful functional status tools such as the New York Heart Association functional status (NYHA FC), Kansas City Cardiomyopathy questionnaire, and 6-min walk test can be utilized to track disease progression and monitor the well-being of patients [62, 63, 75]. Non-molecular biomarkers offer substantial benefits owing to their ease of accessibility and seamless integration into standard healthcare protocols, rendering them a practical and feasible option.

## Electrocardiography

A 12-lead electrocardiogram (ECG) is recommended for diagnostic workup in patients with PPCM, as it is inexpensive, safe, and widely available.[5, 61–63]. Tibazarwa and colleagues reported that more than 90% of PPCM patients at presentation have non-specific ECG abnormalities, which include sinus tachycardia, aberrant conduction, commonly left bundle branch block (LBBB), atrial fibrillation, repolarisation, abnormalities (T wave inversion), and prolonged corrected QT interval (QTc) [76, 77]. In a cohort of 66 PPCM patients, Hoevelmann and colleagues reported that sinus arrhythmia at diagnosis was associated with good outcomes [78]. On the contrary, sinus tachycardia and prolonged QTc interval at presentation were noted to be a predictor of poor prognosis [77–79].

## Cardiac imaging

Cardiovascular imaging in PPCM is classified as non-ionizing (echocardiography) and ionizing (chest radiography (CXR), cardiac magnetic resonance (CMR), computed tomography (CT), ventilation/perfusion (V/Q) scan, invasive coronary angiography (ICA), and nuclear medicine imaging (single-photon emission computed tomography (SPECT)/positron emission tomography (PET) [5, 80]. Multi-modality cardiovascular imaging plays a crucial role in the diagnosis, risk stratification, prognostication, and therapeutic guidance on follow-up of PPCM (Table 2) [81].

**Table 2:** Approach to cardiac imaging in peripartum cardiomyopathy

	Imaging modality	Utility
Initial evaluation	Transthoracic echocardiography (TTE)	First-line for assessing cardiac structure and function • Assessment of left ventricular function: Measurement of left ventricular ejection fraction (LVEF) is crucial to diagnose and assess the severity of PPCM • Chamber sizes and wall thickness: Evaluate the size of the cardiac chambers and wall thickness, which can indicate dilated cardiomyopathy • Diastolic function: Assesses diastolic dysfunction, which can be present in PPCM • Regional wall motion abnormalities: Identifies specific areas of the heart that may not be contracting properly • Valve function: Evaluates for any concomitant valvular abnormalities that may complicate PPCM Speckle tracking echocardiography: • Global longitudinal strain (GLS): Provides early detection of subclinical myocardial dysfunction, which may not be evident on conventional TTE
	Chest X-ray	To assess for cardiomegaly and pulmonary congestion
Further assessment and characterization	Cardiac magnetic resonance	It is a valuable adjunct to TTE, especially if images are suboptimal or detailed tissue characterization is needed to identify fibrosis, inflammation, or other myocardial changes Quantification of ventricular volumes and function: • Provides precise measurements of ventricular volumes, LVEF, and myocardial mass Myocardial tissue characterization: • Late gadolinium enhancement (LGE): Identifies areas of myocardial fibrosis or scarring • T1 and T2 mapping: Detects myocardial oedema and fibrosis, helping to understand the extent and nature of myocardial involvement
Follow-up and monitoring	Serial echocardiography	To monitor changes in ventricular size, function, and recovery over time
	Cardiac magnetic resonance	It may be repeated if there are changes in clinical status or if a detailed re-assessment is needed
Exclusion of other conditions	Coronary CT angiography or Invasive coronary angiography	If there is suspicion of ischemic heart disease contributing to the clinical picture

## Non-ionizing cardiac imaging

### *Echocardiography*

Echocardiography refers to all cardiac ultrasound imaging techniques and is the preferred imaging modality in PPCM as it is widely available [5, 15, 30, 61]. Transthoracic echocardiography (TTE) is cheap, safe during pregnancy and lactation, reproducible, and provides real-time cardiac structural and functional assessment necessary to diagnose PPCM promptly [72, 82]. Normal pregnancy adaptive structural changes include mild chamber dilation, LV wall thickening, elevated cardiac output, and increased LV filling pressures. LV systolic dysfunction with an ejection fraction of < 45% with or without chamber dilation is a criterion for the current diagnosis of PPCM [5]. Complications such as functional mitral regurgitation, pulmonary hypertension, and intramural thrombus can be identified in TTE [16, 19, 83]. Myocardial strain analysis, notably global longitudinal strain (GLS), has been validated as a diagnostic and prognostic parameter in cardiac failure [84, 85].

Kiran and colleagues, in a cohort of 43 PPCM patients, reported low right ventricular (RV) fractional area change (RVFAC 31.4% with 86% accuracy) and high left atrial volume index (LAVi > 29.6 ml/m^2^, with 72% accuracy) at presentation to be independent predictors of adverse events [86]. The EORP PPCM registry confirmed RV dysfunction’s prognostic significance [87, 88]. In addition, LVEF < 30% and LV dilation (left ventricular end-diastolic diameter (LVEDD) ≥ 60 mm) are associated with poor outcomes [87, 89].

## Cardiac magnetic resonance (CMR)

Cardiovascular magnetic resonance imaging is the gold standard radiation-free modality for assessing cardiac structure and function and myocardial tissue characterization [90]. The main advantage is that CMR has a higher spatial and temporal resolution, outstanding accuracy, reproducibility, independence from the acoustic window, and higher sensitivity for detecting LV thrombus [80, 90–93]. Cardiac MRI is an excellent technique for ruling out other de novo heart failure diagnoses in the peripartum period to complement echocardiography, especially in cases with inconclusive or uncertain results [5, 15, 94]. Late gadolinium enhancement (LGE) signifies myocardial fibrosis or scarring. It is usually found in the mid-myocardial or subepicardial segments on the lateral wall but can also be present in other areas in a diffuse or patchy pattern in PPCM. LGE can help rule out diagnoses such as myocarditis [19, 81, 95, 96]. In a cohort of 10 patients, Arora and colleagues reported that LGE was associated with poor outcomes [91]. CMR is not widely available and is expensive. There is a shortage of clinicians with interpretation expertise, and gadolinium-based studies should be avoided during the first trimester [92]. In PPCM, though CMR offers unparalleled insights into the cardiac structure and function, aiding in risk stratification, confirming the diagnosis, prognosticating and monitoring therapeutic success, it is currently not used in routine clinical practice [92–94].

## Ionizing cardiac imaging

### Chest radiography (CXR)

Chest radiography is one of the most accessible and reproducible initial investigations in PPCM [82]. Notable radiological changes include cardiomegaly and pulmonary infiltrates varying with the degree of pulmonary venous hypertension, depicting the severity of mean capillary wedge pressure. In PPCM, the utility of CXR at presentation supports the diagnosis, stratifies the extent of heart failure, and excludes other acute chest syndromes [5].

## Computer tomography (CT)

Cardiac tomography is not the first-line imaging modality for evaluating peripartum cardiomyopathy (PPCM) but can be used for this purpose [5]. Its role includes enhancing echocardiography when the diagnosis is unclear, assessing cardiac structure and function, and detecting intracardiac and extracardiac complications such as intramural thrombus and pulmonary embolus [80, 81]. Furthermore, it can be used to assess coronary artery disease and aid in distinguishing PPCM from other types of cardiomyopathies and myocarditis by providing detailed images of myocardial tissue characteristics. Cardiac CT has advantages due to its non-invasiveness, reproducibility, independence from poor acoustic windows, and high spatial resolution. It can be beneficial in cases where surgical intervention is necessary by providing detailed anatomical information. If necessary, iodinated contrast agents can be used during pregnancy and breastfeeding. However, it is important to note that computer tomography is seldom used in routine PPCM care bundles due to radiation and cost [5, 18, 80]. Transthoracic echocardiography (TTE) and cardiac MRI are often preferred because they do not involve radiation and can provide detailed functional and structural data sets [81].

Although cardiac CT is not the primary imaging method for peripartum cardiomyopathy, it can be valuable in specific clinical situations requiring detailed anatomical information or when complications are suspected. Its use should be carefully considered due to the risks of radiation exposure, especially during the peripartum period [81].

## Nuclear imaging

Nuclear imaging techniques such as single-photon emission computed tomography (SPECT), positron emission tomography (PET), and cardiac scintigraphy or myocardial perfusion imaging (MPI) have limited use in diagnosing PPCM [5]. They are mainly used to distinguish PPCM from ischaemic cardiomyopathy [80, 81]. A ventilation/perfusion (V/Q) scan is particularly useful in identifying or ruling out pulmonary embolism, which has similar symptoms to PPCM. V/Q scans involve lower radiation exposure compared to CT pulmonary angiography, making them safer for postpartum women [80]. However, it is important to note that even low doses of radiation exposure can be concerning, especially during pregnancy. Additionally, these techniques can be costly and require expertise in nuclear cardiology to accurately interpret results [81].

Although it has limitations, nuclear imaging can be beneficial in certain situations for thoroughly evaluating PPCM. It can be crucial in ruling out conditions like pulmonary embolism and coronary artery disease, which have symptoms similar to PPCM. This ensures that PPCM can be managed appropriately and effectively.

## Endomyocardial biopsy

Endomyocardial biopsy is not routinely used to diagnose and predict PPCM unless a cardiac transplant is considered. It is useful when myocarditis or other rare metabolic or storage cardiac myocyte diseases are suspected [5, 93].

The use of molecular and non-molecular biomarkers in PPCM provides valuable information for diagnosing, prognosticating, and managing disease. Cardiac biomarkers like BNP/NT-proBNP and troponins play a pivotal role in diagnosis, while inflammatory, oxidative stress and prolactin-related markers offer insights into the disease’s mechanisms and potential treatment options. These biomarkers’ comprehensive comprehension and application can significantly enhance patient care and outcomes in PPCM.

## Management

The clinical management of PPCM is a daunting task without disease-specific, evidence-based data; the therapeutic strategy mirrors other forms of HF with left ventricular systolic dysfunction directed by international guidelines [5, 15–19, 61–63, 97]. A multi-disciplinary approach is crucial as PPCM has peculiarities and nuances necessitating the concerted input from cardiologists, obstetricians, obstetric-medicine physicians, intensivists, cardiac surgeons, anaesthesiologists, neonatologists, and nurses [5, 15, 96–98]. In stable PPCM, Bauersachs and colleagues recommend the BOARD scheme therapeutic approach (bromocriptine, oxygen, anticoagulation, relaxants, and diuretics) [99].

Most patients present with de novo decompensated HF, characterized by exertional dyspnea that eventually progresses to rest dyspnea and fluid overload [61]. Generally, mechanical ventilatory and circulatory support may be required in cardiopulmonary compromise with inotropic support [5, 15, 61–63]. Urgent delivery with caesarean section should be instituted [82].

The peripartum period is highly oxidative, and STAT3 expression is protective [44]. When inotropic support is required, noradrenaline and levosimendan are preferred to dobutamine, a beta 1-adrenergic receptor agonist. Dobutamine is controversial as it has been associated with worse outcomes due to persistent beta 1-adrenergic receptor activation [5, 15, 100, 101]. Stepal and colleagues noted that chronic beta 1-adrenergic receptor stimulation was associated with heart failure in both postpartum and in mice with cardiomyocyte-specific STAT3 depletion and a proof of concept in the German registry cohort [101].

**Fig. 3:**
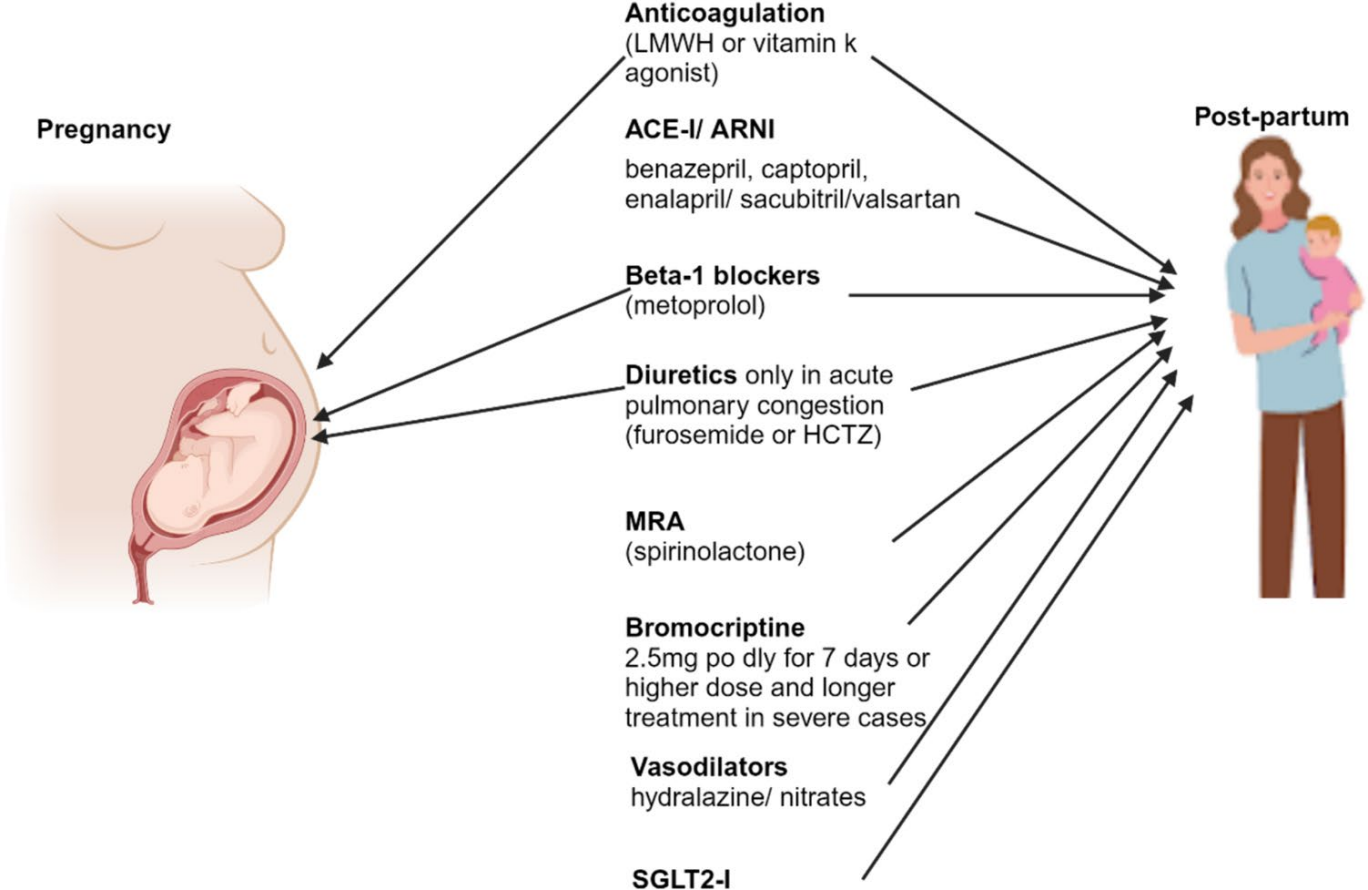
Pharmacological therapy in peripartum cardiomyopathy. Drug therapy of PPCM during pregnancy includes the following: beta-1 receptor selective blockers (metoprolol is preferred while atenolol should be avoided); diuretics such as furosemide and hydrochlorothiazides should be used only in the presence of pulmonary congestion as they may decrease blood flow to the placenta; and anticoagulation therapy such as LMWH or vitamin k antagonists can be used at prophylactic dose or at therapeutic dose in the presence of intracardiac or systemic thrombo-embolism according to the stage of pregnancy. ACE-I, ARNI, and MRA are contraindicated during pregnancy. Drug therapy post-partum includes the aforementioned therapies in addition to ACE-I/ARNI, MRA, vasodilators, SGLT2-I, and bromocriptine. ACE-I, angiotensin-converting enzyme inhibitor; ARNI, angiotensin receptor/ neprilysin inhibitor; HCTZ, hydrochlorothiazide; LMWH, low-molecular-weight heparin; MRA, mineralocorticoid receptor antagonist; SGLT2-I, sodium-glucose cotransporter-2 inhibitors. Created with BioRender.com

In chronic PPCM in the postpartum period, optimal guideline-directed medical therapy (GDMT) consists of the four heart failure pillars of disease-modifying drugs, namely renin–angiotensin–aldosterone (RAAS) inhibitors (angiotensin converting enzyme inhibitors (ACEI)/ angiotensin receptor/neprilysin receptor inhibitors (ARNI) and mineralocorticoid receptor antagonist (MRA)), sodium-glucose cotransporter-2 (SGLT2) inhibitors, and beta-blockers (Fig. 3) [15, 61–63, 82]. The historic hierarchical introduction of HF therapy is an area of guideline development, as a rapid commencement of all four drug classes is recommended, preferably before hospital discharge [96–98]. Additionally, ivabradine, vericiguat, hydralazine, and isosorbide dinitrate can be added to select PPCM patients [61–63, 96]. RAAS inhibitors are not recommended during pregnancy due to fetotoxicity. Instead, a combination of hydralazine and nitrates may be used.

SGLT2 inhibitors, ivabradine, and vericiguat are not recommended during pregnancy and in breastfeeding mothers due to the paucity of safety data [16, 19, 96]. Advanced heart failure therapies in PPCM include cardiac resynchronisation therapy, implantable cardioverter defibrillator, left ventricular assist device, and cardiac transplant. These therapies are commonly used in high-income countries but are not readily available in middle- and low-income countries, even though these countries bear the most significant disease burden [12, 16, 96]. Anticoagulation is usually recommended in patients with intramural thrombus, venous thromboembolic disease, and atrial fibrillation. In PPCM patients with very low EF, prophylactic anticoagulation should be considered [5, 37, 82, 99]. Wearable cardiac defibrillators are recommended for newly diagnosed PPCM with LVEF < 35% for risk of sudden cardiac death due to ventricular tachycardia [5].

Bromocriptine is a dopamine agonist that blocks prolactin production. The drug is recommended in PPCM patients with severe left ventricular systolic dysfunction or those unwilling to breastfeed [9, 61, 82]. Based on evidence from recent studies, the ESC HF guidelines recommend adding bromocriptine to treat acute PPCM, especially in severe cases. However, the AHA/ACC HF guidelines have not approved using bromocriptine in PPCM because it is not FDA-approved for that purpose [5, 15–19, 51, 96, 102–106]. Patients taking bromocriptine are advised to take preventative anticoagulants because bromocriptine is pro-thrombotic (Regitz) [82–107]. The ongoing REBIRTH (Impact of Bromocriptine on Clinical Outcomes for Peripartum Cardiomyopathy) study in North America is designed to address the safety and efficacy of bromocriptine [5, 17].

It is recommended that all patients diagnosed with peripartum cardiomyopathy (PPCM) undergo genetic testing if resources permit, especially those with a genetic predisposition. This includes patients with a family history of PPCM, a known cardiomyopathy mutation, or specific ethnic backgrounds. The process involves genetic counselling, DNA extraction, targeted sequencing of known cardiomyopathy-related genes, and genome or exome sequencing. Subsequently, any identified mutations are interpreted. Individuals with positive genetic results should undergo post-test genetic counselling, and their relatives should be screened to identify at-risk individuals who may require cardiac follow-up [56].

## Patient education

**Fig. 4:**
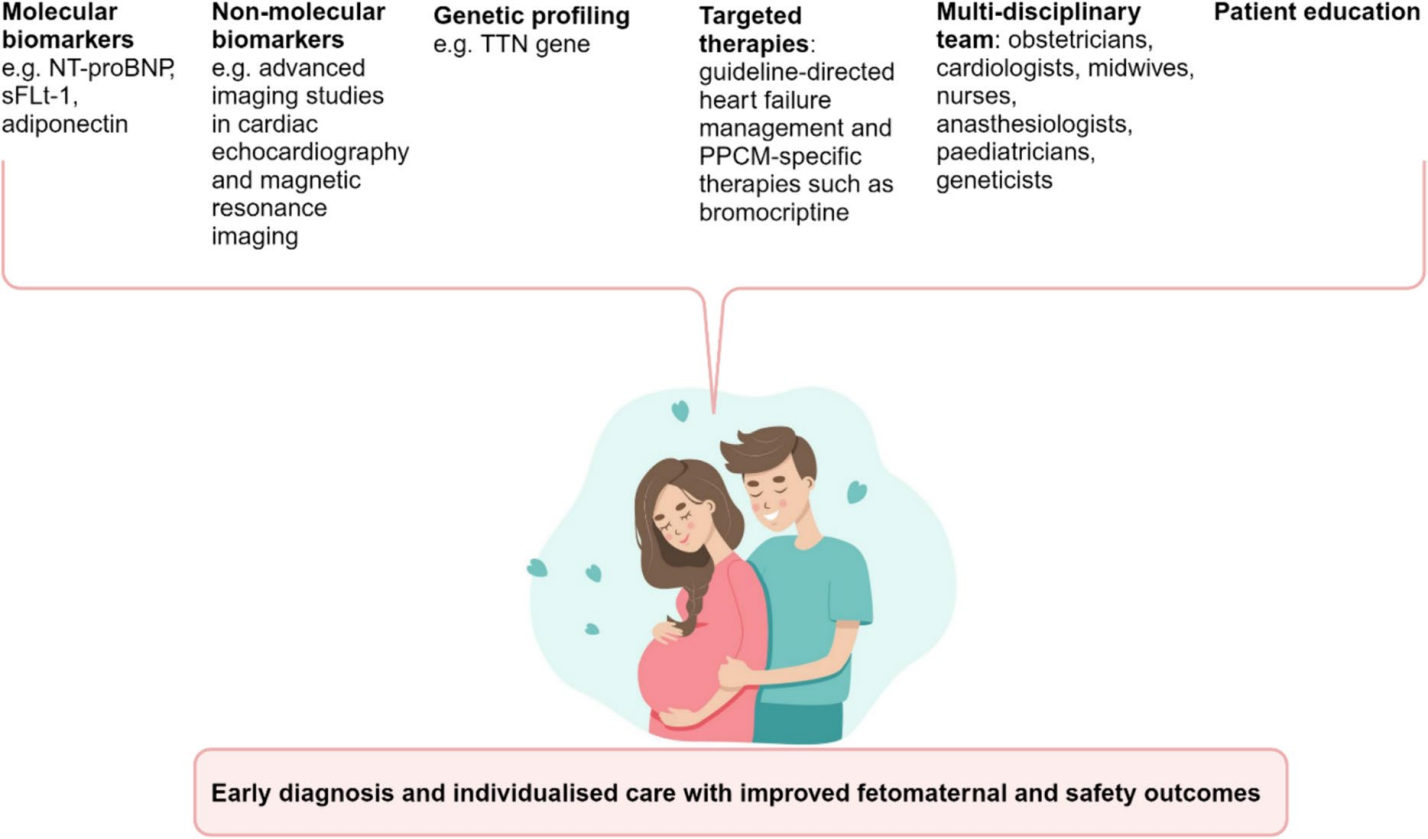
The integrated care of peripartum cardiomyopathy. The integration of biomarkers, genetic profiling, conventional and disease-specific guideline-directed therapy, patient education, and a multi-disciplinary team contributes to early diagnosis and individualized care of peripartum cardiomyopathy, which leads to improved fetomaternal and safety outcomes. NT-proBNP, N-terminal pro b-type natriuretic peptide; sFLt-1, soluble Fms-like tyrosine kinase 1; PPCM, peripartum cardiomyopathy; TTN, titin protein. Created with BioRender.com

Patient education is crucial for the integrated management of PPCM for several reasons (Fig. 4) [5, 108–111]. It raises awareness, enabling early detection of medical conditions by imparting knowledge about risk factors, medication adherence, and disease monitoring. Moreover, it facilitates patients’ access to support groups and resources for family planning, compliance with follow-up care, and adoption of pregnancy precautions [109, 110]. However, a pressing need exists for enhanced utilization and further research on patient education in PPCM management. The establishment of cardiac-obstetric clinics staffed with proficient multi-disciplinary healthcare professionals who are well-versed in the intricacies of PPCM is imperative [98]. Patient education empowers women to make well-informed decisions about their health, facilitate self-monitoring of their condition, adhere to treatment plans, and seek timely medical care as needed [5, 61–63, 110]. This underutilized resource is pivotal for delivering comprehensive care and support to individuals affected by PPCM.

## Novel therapeutic targets

Peripartum cardiomyopathy management has a tremendous unmet need for active research and investigation into potential therapeutic targets [5, 71, 75, 112–115]. In the dynamic field of PPCM research, it is crucial to recognize that while numerous therapeutic targets have shown promise in early studies, only some of these therapies are guaranteed to translate from the bench to the bedside [71]. Table 3 illustrates the potential novel therapeutic targets in PPCM [113–121].

**Table 3:** Potential therapeutic targets in peripartum cardiomyopathy

Therapeutic target	Molecular property	Potential benefit in PPCM
Ivabradine	Reduces heart rate through selective inhibition of the funny current (If) in the sinoatrial node	Reduction in workload on the heart and improvement of cardiac function
MicroRNA-146a antisense	Regulates inflammation and immune responses	Potential reduction in inflammation and improvement of cardiac function
Adiponectin	Holds pleiotropic metabolic effects, including potential benefits for cardiovascular health	Research is ongoing regarding its use as a therapeutic target of PPCM
Perhexiline	Prevents angina, thus improving myocardial energetics switching from fatty acids β oxidation to glycolysis	Potential improvement of cardiac function by improving the heart’s energy utilization
VEGF analogues	Promotes the production of new blood vessels	Promotion of better blood supply to cardiomyocytes
Seralaxin	Recombinant Relaxin-2, vasodilatory and anti-fibrotic effects	Potential treatment for heart failure, including PPCM
Pentoxyfylline	Anti-inflammatory properties	Prevention of inflammation associated with PPCM
Liver x receptor/ Retinoid x receptor (LXR/RXR)	Regulation of cholesterol metabolism-mediated anti-inflammation	Enhanced PPCM recovery through upregulation of LXR/RXR signalling

*MicroRNA-146a* microribonucleic acid-146a, *PPCM* peripartum cardiomyopathy, *VEGF* vascular endothelial growth factor

Serum proteomic profiling (SPP) in PPCM is an emerging area of research that aims to identify specific protein biomarkers associated with the disease by analyzing the protein composition of blood samples to uncover unique patterns, signatures, or themes that may help in diagnosis, understanding pathophysiology, and guiding treatment strategies [113, 114].

A study conducted by Kodogo et al. utilized an untargeted SPP to analyze patients diagnosed with PPCM. This study is significant as it revealed 15 upregulated and 14 downregulated proteins, establishing a significant association between PPCM and a combination of adiponectin, quiescin sulfhydryl oxidase 1, inter-α-trypsin inhibitor heavy chain, and NT-proBNP [113]. These findings indicate salient biological themes related to immune response, inflammation, and coagulation and provide valuable insights into the pathophysiology of PPCM. Furthermore, Lovell and colleagues observed analogous perturbations in inflammation and lipid metabolism utilizing proteomic methodologies, further validating the findings of the Kodogo study [114]. These advancements have unveiled the intricate and diverse mechanisms underlying the pathophysiology of PPCM, warranting further research.

## Clinical outcomes

### Maternal outcomes

PPCM is potentially life-threatening if not diagnosed early and managed appropriately [5, 61]. Heart failure can be progressive, leading to reduced effort tolerance, poor quality of life, and even death. Maternal complications include hypoxia, thromboembolism, arrhythmias, hospital readmission, and misdiagnosis as preeclampsia [5, 110]. Traditionally, maternal outcomes were known to follow the rule of thirds, where a third recovered, remained stable, and deteriorated [22]. In recent years, the increased disease awareness combined with the advent of HF anti-remodelling therapy improved outcomes, with nearly half recovering, a quarter remaining stable, and another quarter eventually deteriorating [19, 122–128]. In PPCM patients on GDMT who fully recover, the continuation of anti-remodelling therapy is recommended [5, 124]. Halliday and colleagues, in a pilot trial, though underpowered, showed that in heart failure patients with improved or recovered ejection fraction, 40% relapsed on discontinuation of therapy [11].

Counselling regarding subsequent pregnancies is of utmost importance for all PPCM patients, emphasizing the potential for relapse, exacerbation of heart failure, and mortality, notwithstanding an improved or recovered ejection fraction [109–111]. The treatment armamentarium for heart failure has undergone a paradigm shift with the introduction of SGLT2 inhibitors [62, 63, 98]. This advancement is anticipated to enhance further the clinical outcomes of peripartum cardiomyopathy (PPCM). Further investigation is necessary to examine the clinical outcomes of patients undergoing novel and robust heart failure therapy.

### Fetal outcomes

Fetal complications may be due to fetal distress secondary to maternal hypoxia or placental hypoperfusion caused by reduced cardiac output induced by excessive diuresis or reduction in blood pressure from aggressive afterload reduction [122–124]. Prematurity, stillbirth, and neonatal death are also among some of the fetal complications [126]. There is a lack of data on neonatal outcomes in patients with PPCM, as they are seldom reported [5–7, 126].

## Predictors of clinical outcome

In the initial 6 months following the diagnosis of PPCM, ventricular remodelling occurs alongside an exponential recovery of left ventricular function [89, 90]. The role of baseline LVEF and dimension as predictors of mortality has been a subject of debate, with conflicting findings across various studies [84]. Previous studies might have failed to demonstrate significant differences due to insufficient data or statistical power to establish a clear association between baseline LVEF or dimension and mortality in PPCM patients [86, 128]. However, recent research suggests that these baseline parameters may hold predictive value in predicting outcomes in PPCM [67, 72, 76, 84]. Higher NYHA functional class, Fas/Apo-1, prolonged hospitalization and admission to the intensive care unit (ICU) at diagnosis were linked to increased mortality risk [86]. At the same time, novel findings have identified young age (< 20 years) and extreme body mass index (< 18.5 kg/m^2^ and > 30 kg/m^2^) at diagnosis as independent predictors of mortality [13, 16, 126]. Hoevelmann and colleagues observed that prolonged QTc and sinus tachycardia at baseline on ECG also predict poor outcomes among PPCM patients at 6 and 12 months, respectively [78]. Several contemporary studies have highlighted RV dysfunction and reduced LV global longitudinal and circumferential strain patterns as indicators of adverse PPCM prognosis [14, 129]. Patients with a history of previous PPCM are susceptible to relapse or deterioration during SSP [7, 42, 125, 128, 130–132]. In a cohort of 34 SSP PPCM patients, Hilfiker-Kleiner and colleagues reported an overall relapse rate of 53% and a mortality rate of 12% [132]. The study also noted that persistently reduced LV systolic function was associated with high mortality and a lower recovery rate. In patients with recovered systolic function, exercise stress tests with adequate contractile reserve may identify individuals at low risk of PPCM in subsequent pregnancies. Currently, novel risk prediction scores are being developed for PPCM to identify high-risk individuals, expedite diagnosis, forecast outcomes, and provide guidance for effective management [133–135].

## Conclusion

Peripartum cardiomyopathy (PPCM) is a potentially life-threatening condition, but its global prevalence and incidence are still unknown due to misdiagnosis and underreporting. The pathophysiology of PPCM is complex, and it remains a diagnosis of exclusion despite increased research. The only well-established biomarkers for diagnosis and guiding therapy are natriuretic peptides pro-BNP/NT-proBNP. However, other potential biomarkers such as sFlt, 16 kDa PRL, micro-RNA, SPP, and multi-modality cardiac imaging require further research. Clinical management follows conventional heart failure therapy, with recent focus on ARNI and SGLT2 inhibitors. Bromocriptine is currently the only disease-specific therapy, with additional efficacy and safety studies underway. Most women with the integration of PPCM care recover, and future research should concentrate on identifying disease-specific biomarkers and developing risk stratification models.

## Data Availability

Not applicable—the current paper is a review.

## Abbreviations

AHA/ACC: American Heart Association/American College of Cardiology
CVD: Cardiovascular disease
ESC: European Society of Cardiology
HF: Heart failure
LV: Left ventricle
LVEF: Left ventricular ejection fraction
MnSOD: Manganese superoxide dismutase
NYHA: New York Heart Association
PGC1ᾳ: Peroxisome proliferator-activated receptor gamma 1-alpha coactivator
PPCM: Peripartum cardiomyopathy
PRL: Prolactin
ROS: Reactive oxygen species
RV: Right ventricle
sFlt1: Soluble fms-like tyrosine kinase-1
SGLT2i: Sodium-glucose cotransporter-2 inhibitors
SPP: Serum proteomic profile
SSP: Subsequent pregnancy
STAT3: Signal transducer and activator of the transcription 3
VEGF: Vascular endothelial growth factor
